# The Effect of Experience on Visual Search Patterns in Retinal Imaging Analysis

**DOI:** 10.3928/23258160-20250228-03

**Published:** 2025-03-01

**Authors:** Puranjay Gupta, Neil Sheth, Reem AlAhmadi, Xincheng Yao, Michael J. Heiferman

**Affiliations:** Department of Ophthalmology and Visual Sciences, University of Illinois College of Medicine at Chicago, Chicago, Illinois; Department of Ophthalmology and Visual Sciences, University of Illinois College of Medicine at Chicago, Chicago, Illinois; King Khaled Eye Specialist Hospital, Riyadh, Saudi Arabia; Department of Ophthalmology and Visual Sciences, University of Illinois College of Medicine at Chicago, Chicago, Illinois; Department of Ophthalmology and Visual Sciences, University of Illinois College of Medicine at Chicago, Chicago, Illinois

## Abstract

**BACKGROUND AND OBJECTIVE:**

The increasing use of retinal diagnostic imaging necessitates a standardized viewing technique. This study investigates visual search patterns among ophthalmologists at various experience levels using eye-tracking technology.

**PATIENTS AND METHODS:**

Participants included postgraduate year 2, 3, and 4 residents, retina fellows, and attending ophthalmologists, who analyzed fundus images while their eye movements were tracked.

**RESULTS:**

Results indicated that attendings had shorter fixation durations (0.15 ± 0.04 seconds) and saccade lengths (0.06° ± 0.01°), indicating faster image information processing than novice physicians. Experts also analyzed a higher proportion of the image area (49.43% ± 7.34%) and possessed a global-focal search pattern, suggesting increased thoroughness.

**CONCLUSION:**

Experts in ophthalmology demonstrate gaze characteristics that reflect faster image processing and a more thorough analysis of diagnostic imaging. We recommend that residents be taught a standardized method for image interpretation that emulates expert analysis through a disc-macula-vessel-periphery sequence with radial sweeps.

Digital retinal imaging has become an increasingly integral part of the diagnostic workup and therapeutic monitoring of retinal disorders with the development of new imaging modalities.^1,2^ Unlike many other medical specialties, ophthalmologists order and interpret retinal imaging themselves. Medical specialties with a more central focus on visual interpretation, such as radiology and pathology, have extensive literature studying the process.^3–5^ Retinal imaging presents unique challenges for which there is a current paucity of literature.

Visual search patterns describe the eye movements and fixations of the viewers as they examine an image. Two common characteristics of search patterns studied are fixations and saccades, the movements between fixations. Research in radiology and pathology has shown significant differences between the visual search patterns of experts and trainees. Attendings are significantly more efficient at surveying images and extracting information than residents and medical students.^3,6^ Furthermore, there are noted improvements in image analysis during training, where pathology residents increase their efficiency after their first year and between their third and fourth years of residency.^5^

Organized visual search patterns, such as the disc-macula-vessel-periphery sequence, are commonly employed in examining retinal imaging. However, there is limited empirical evidence supporting the efficacy of these standardized approaches. Residents are taught to evaluate retina imaging during fluorescein conferences, starting with the systematic description of the imaging modality and eye laterality, followed by a search pattern to evaluate the media, optic disc, macula, vessels, and retinal periphery methodically. The American Academy of Ophthalmology describes a systematic approach to evaluating rhegmatogenous retinal detachment by assessing the configuration, location, and extent of the detachment, identifying and characterizing retinal breaks, and noting macular involvement, posterior vitreous detachment (PVD), and proliferative vitreoretinopathy (PVR) changes.^7^

Understanding the differences in visual search patterns between trainees and experts also has significant educational implications for ophthalmology. This study can help refine current training methods and improve diagnostic accuracy by identifying novel search patterns. Eye-tracking technology has proven effective in laparoscopy, where projecting a supervisor’s gaze data onto surgical tasks improved completion times and accuracy.^8^ Applying similar eye-tracking techniques in ophthalmology could potentially offer trainees real-time feedback and help them develop expert-level search strategies.

Therefore, with the lack of existing literature investigating the visual search patterns of experts and trainees, we hypothesized that differences in search patterns exist between ophthalmology groups of varying expertise and that an increase in expertise would coincide with an increase in efficient and accurate image processing.

## METHODS

We performed an institution review board-approved, observational study of ophthalmology trainees and faculty recruited at the Department of Ophthalmology and Visual Sciences, University of Illinois College of Medicine at Chicago. Data collection was performed from October 2022 to December 2024. Participants were recruited at each experience level: postgraduate years 2, 3, and 4 (PGY2, PGY3, PGY4) ophthalmology residents, vitreoretinal surgery fellows (fellows), and attending vitreoretinal surgeons (attendings), with five participants in each group (*n* = 5). Each group had participants with similar years of experience in diagnosing retinal images (Table 1). The participants provided a diagnosis from 13 ultra-widefield fundus images taken on a California Ultra-widefield Retinal Imaging System (Optos). These images were sourced from a de-identified database at the Department of Ophthalmology and Visual Sciences, University of Illinois College of Medicine at Chicago, and therefore, no patient demographic information was available. For consistency, participants were instructed to assume the images were from individuals aged 50 with no known medical history. The fundus images comprised 14 retinal pathologies plus one normal retina. These images are shown in Figure A, available with this article online, with the pathologies labeled. During the study, participants viewed the same images without these labels.

The following procedures are derived from studies using pupil tracker systems as an independent visual diagnostic performance assessment tool.^9–11^ The image analysis setup consisted of a monitor keyboard with images in full-screen slide format. The subjects could control the pace of the randomly ordered stimulus images via the keyboard. The participants were instructed to read a verbal prompt to confirm proper calibration. Then, a short verbal diagnosis (no longer than 2 to 3 words) regarding each retinal imaging was collected anonymously. At the end of each task, the recordings of the image analysis were saved into a deidentified database, and the masked investigators accessed the database to perform analysis.

The mobile eye-tracking device employed in this study is a Pupil Core eye-tracker (Pupil Labs). Two infrared cameras monitor the eyes at 200 frames per second (FPS) with a resolution of 192 × 192 pixels. The Pupil Capture software analyzes the eye camera data and generates a three-dimensional (3D) eyeball model to determine pupil position. Additionally, the device features a third outward-facing scene camera that captures the participant’s point-of-view (POV) at 30 FPS with a resolution of 1280 × 720 pixels and a diagonal field of view (FOV) of 100°. This scene camera contextualizes gaze data by recording the visual stimuli being viewed and providing an overlay of gaze position. The Pupil Player software was then used to export and analyze the recordings.

Studies have validated the Pupil Core’s effectiveness in various applications. For instance, Velisar and Shanidze assessed measurement uncertainties in head-mounted eye trackers, including the Pupil Core, across multiple metrics.^12^ They found that the Pupil Core System reported consistent and reliable measurement measurements with accuracy ranging between 1.6° and 2.2°, and precision between 0.1° and 0.2°, comparable to other eye-trackers. Additionally, this system has been employed in various medical fields such as diagnostics, telerobotics, and surgical skills assessments to understand the eye movements and behaviors of medical professionals.^13–15^

**Figure 1.**
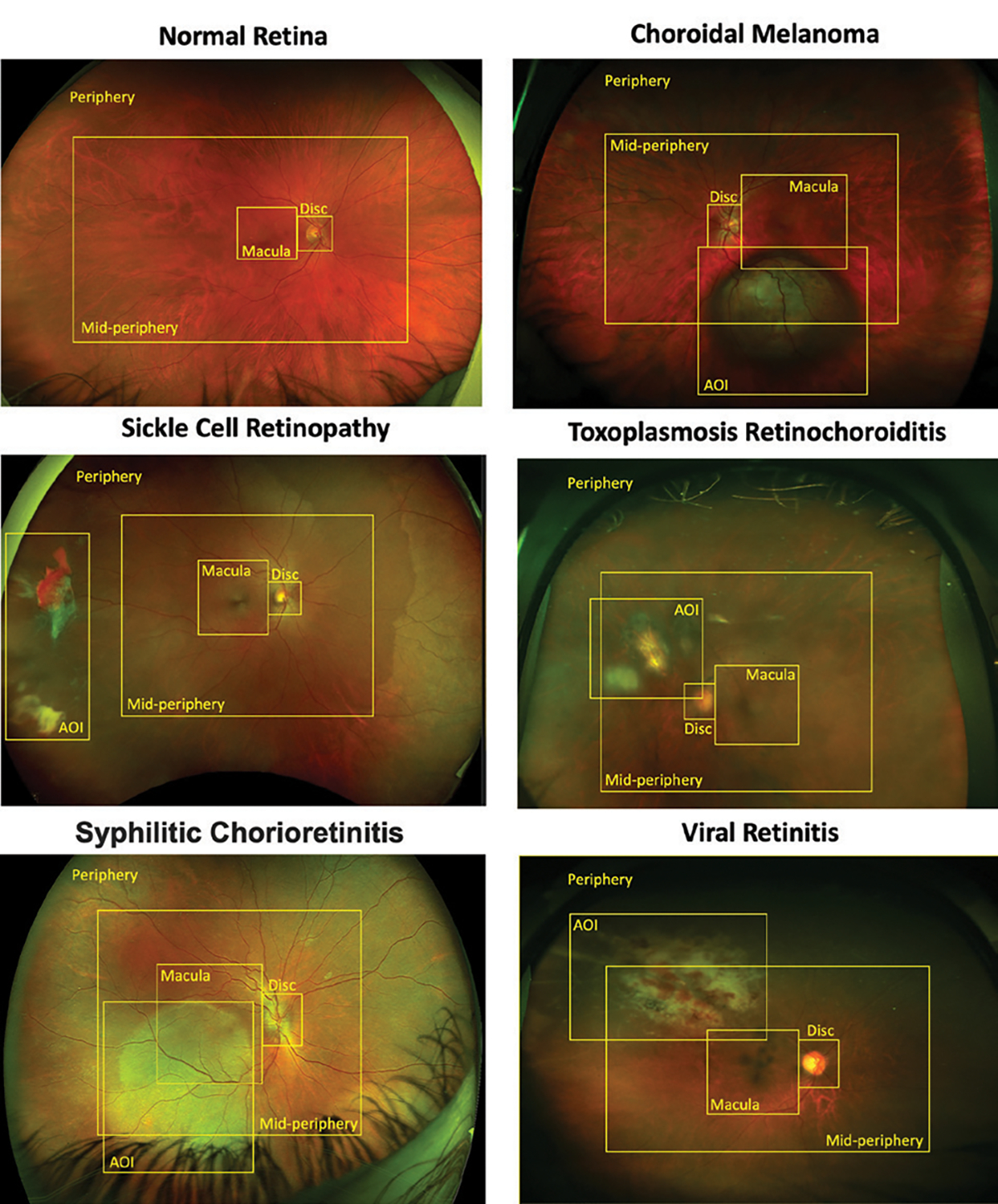
Set of five ultra-widefield images of the fundus (with normal provided as reference) which were selected for the assessment of area of image analysis and the temporal aspects of disc macular vascular peripheral analysis (DMVP). The abnormal retinal images compensate for the participants’ acclimatization to the imaging format, as well as feature focal pathologies to assess analysis patterns.

The analysis focused on two main parameters: fixations and saccades. Fixations represent periods of stable eye movement, indicating attention, while saccades denote rapid eye movements between fixations. Key metrics included number of fixations, fixation durations, blink rates, area of image fixated on, average saccadic amplitude, time spent at each site, and percentage of fixations at each site. Fixations were classified with a maximum dispersion of 1.50°, a minimum duration of 80 ms, and a maximum duration of 220 ms. The area of image analyzed was determined through an image processing algorithm in the Pupil Core system. A 5° circular marker applied a blue mask on top of the baseline image at each x, y, and z position recorded during the trial. Blue pixels (representing covered areas) in the post-trial image were compared against those in the original fundus image to quantify coverage. Five fundus images were chosen for area analysis due to their focal pathologies, defined areas of interest (AOI), as well as disc, macula, vascular (mid-periphery), and periphery regions (Figure 1).

The qualitative observations examined the participants’ fixations to points of interest, image artifacts, and the external environment (outside the monitor). Any qualitative data (eg, pictures and video) was exported in a 16:9 aspect ratio. Data was excluded if the software could not verify its accuracy to 95% confidence. Correlation analyses were performed using the Pearson formula in Origin(Pro), v. 2024 (OriginLab). Correlation results are reported as r (degrees of freedom) = correlation coefficient, *P* = *P* value. A five-group (PGY2, PGY3, PGY4, Fellow, and Attending) analysis of variance (ANOVA) was performed with a post-hoc Tukey’s test (*n* = 5 for all participant groups; 5 groups; α = 0.05). Results from the ANOVA are reported as F(df between groups, df within groups) = F-value, *P* = *P* value. A significance level of α = 0.05 was applied, and *P* values less than 0.05 were considered statistically significant. Results are reported as mean ± standard deviation.

## RESULTS

### ACCURACY OF RESPONSES

**Figure 2.**
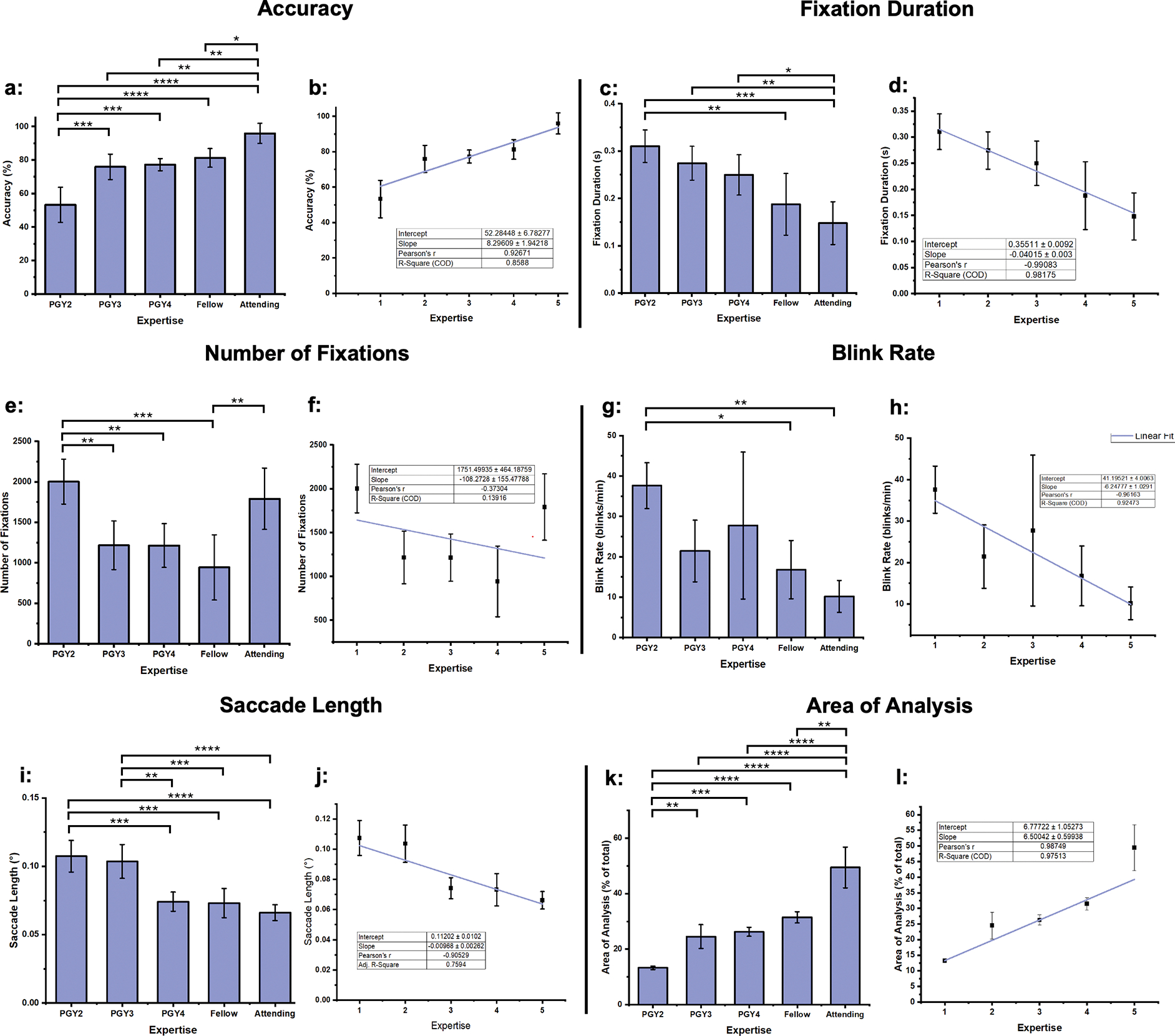
Accumulated data from each of the individual experience levels. (A) Response accuracy. (B) Linear regression of response accuracy relative to experience. Note that experience categories are assigned arbitrary ordinal values, where PGY2 – 1, PGY3 – 2, PGY4 – 3, Fellow – 4, and Attending – 5. This will hold true for the remainder of the figures. (C) Fixation duration. (D) Linear regression of fixation duration relative to experience. (E) Number of fixations. (F) Linear regression of number of fixations relative to experience. (G) Blink rate. (H) Linear regression of blink rate relative to experience. (I) Saccade length. (J) Linear regression of saccade length relative to experience. (K) % area of image fixated on. (L) % area of image fixated on relative to experience. * *P* < 0.05. ** *P* < 0.01. *** *P* < 0.001. **** *P* < 0.0001.

Accuracy of diagnosis was examined to determine the relationship between experience and correct diagnosis. PGY2s demonstrated the lowest proficiency (Figure 2A), with an average score of 53.33% ± 20.54%. Proficiency scores increased with expertise, where attendings achieved significantly higher proficiency scores than PGY2s, averaging 96.00% ± 5.96% F(4,20) = 23.62, *P* < 0.0001. A linear regression analysis (Figure 2B) indicated a significant positive association between the level of medical training and proficiency scores r(3) = 0.93, *P* = 0.02.

### FIXATIONS

The first eye metric examined was fixation duration. The highest average fixation duration was found among the PGY2s, at 0.31 ± 0.034 seconds, while the attendings recorded the shortest average fixation duration, 0.15 ± 0.045 seconds (Figure 2C), significantly different than PGY2s F(2,20) = 10.21, *P* = 0.0016. This pattern is supported by the negative correlation between experience level and fixation duration r(3) = −0.99, *P* = 0.011 (Figure 2D). PGY2s also recorded the highest average number of fixations at 2002 ± 277, followed by attendings with 1791 ± 378 fixations. PGY3s and PGY4s showed similar average numbers of fixations, 1217 ± 300 and 1214 ± 270, respectively, and were both significantly lower than PGY2s F(4,20) = 9.01, *P* = 0.0095, *P* = 0.0093. Fellows recorded the lowest average number of fixations at 1106 ± 463 (Figure 2E). The regression model indicates a poor negative trend r(3) = −0.25, *P* = 0.69 (Figure 2F).

### BLINK RATE

PGY2s exhibited the highest average blink rate, recorded at 37.60 ± 5.67 blinks per minute, while the attendings displayed the lowest average blink rate of all groups at 10.18 ± 3.94 blinks per minute, significantly less than PGY2s, F(4,20) = 5.63, *P* = 0.0024. The more senior PGY4s had a higher blink rate than PGY3s at 21.4 ± 7.6 and 27.7 ± 18.2 blinks per minute, respectively (Figure 2G). Regression analysis indicates a strong negative association between experience and blink rate r(3) = −0.89, *P* = 0.039 (Figure 2H).

### SACCADE LENGTH

The data collected revealed a progressive decrease in average saccade length with increasing medical experience (Figure 2I). PGY2s exhibited the longest saccades with an average length of 0.107° ± 0.011°, diminishing among PGY3s, PGY4s, and fellows. Attendings demonstrated the shortest average saccade length of 0.066° ± 0.0058°, significantly lower than PGY2s’ F(4,20) = 18.82, *P* < 0.0001. The linear regression analysis supported these findings and presented a negative correlation between saccade length and experience level r(3) = −0.93, *P* = 0.020 (Figure 2J).

### AREA OF IMAGE FIXATED

We extended our analysis to the percentage of the image area fixated upon by different experience groups in ophthalmology. PGY2s fixated on the smallest percentage of the image area, averaging 15.24% ± 0.83%. Attendings fixated on the greatest percentage of the image area, with an average of 49.43% ± 7.34%, significantly higher than PGY2s’ F(4,20) = 55.06, *P* < 0.0001 (Figure 2K). The linear regression analysis (Figure 2L) portrayed a significant positive association between experience level and the percentage of image area fixated upon r(3) = 0.95, *P* = 0.014.

### TIME SPENT ANALYZING EACH AREA OF THE IMAGE

**Figure 3.**
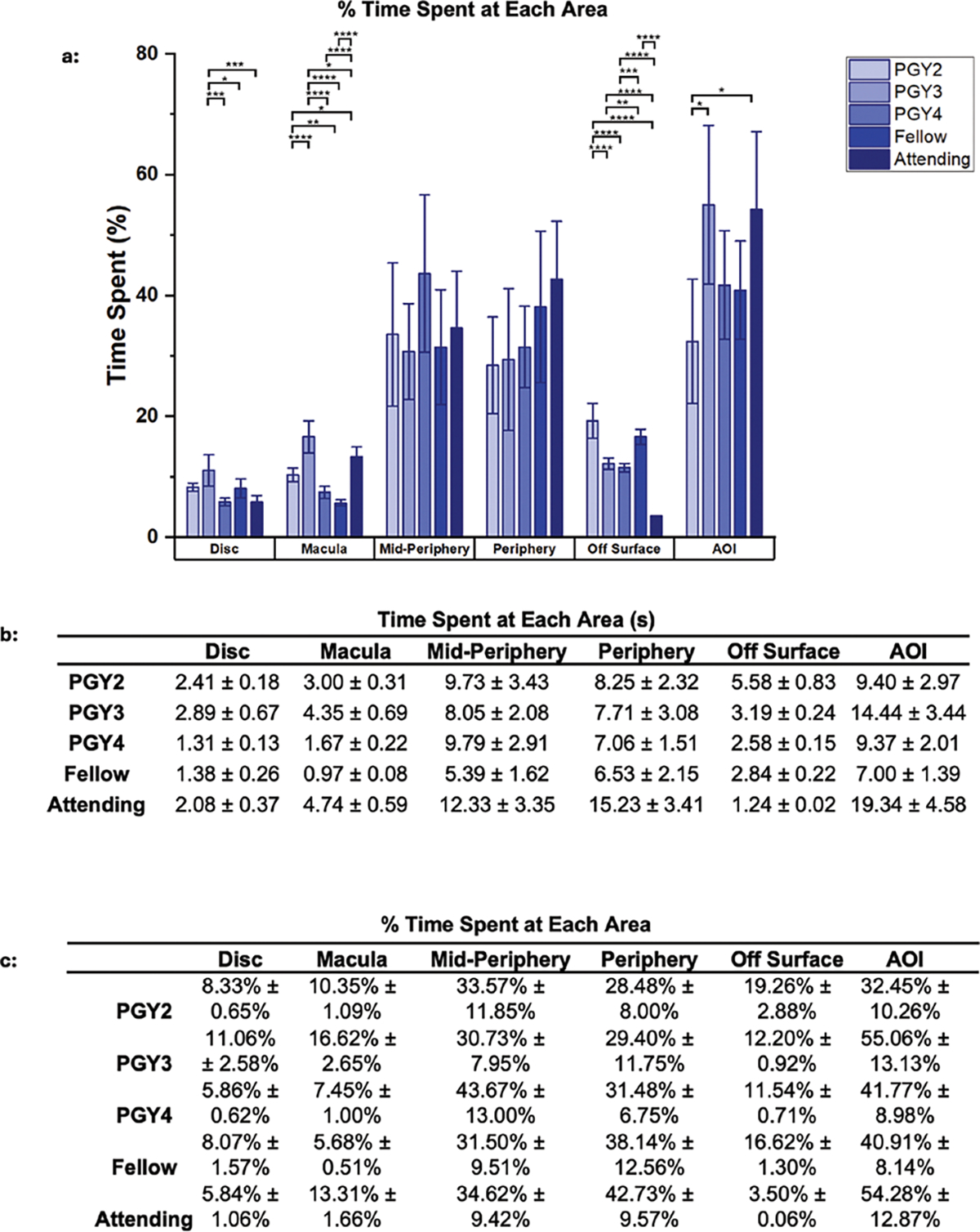
Accumulated data from each of the individual experience levels. (A) Plotted data of the % time spent on analysis in each eye location from the participant groups for between group analysis. (B) Table values of the plotted time data. (C) Table values of the % time spent on analysis in each eye location from the participant groups for between group analysis. * *P* < 0.05. ** *P* < 0.01. *** *P* < 0.001. **** *P* < 0.0001.

We further analyzed the distribution of gaze time within each area of the fundus image by different medical experience levels (Figure 3). Attendings spent an average of 19.34 ± 4.58 seconds on the AOI (54.28% ± 12.87% of their total analysis time), showing significant emphasis on this region. PGY2s allocated 9.40 ± 2.97 seconds (32.45% ± 10.26%) to the AOI. PGY4s spent proportionately the most time analyzing the mid-periphery, with 9.79 ± 2.91 seconds (43.67% ± 13.00%). Attendings, however, displayed a more distributed approach, spending 12.33 ± 3.35 seconds (34.62% ± 9.42%) on the mid-periphery. The macula and off-surface areas were less emphasized by all groups, receiving relatively lower percentages and durations of attention.

### NUMBER OF FIXATIONS ANALYZING EACH AREA OF THE IMAGE

**Figure 4.**
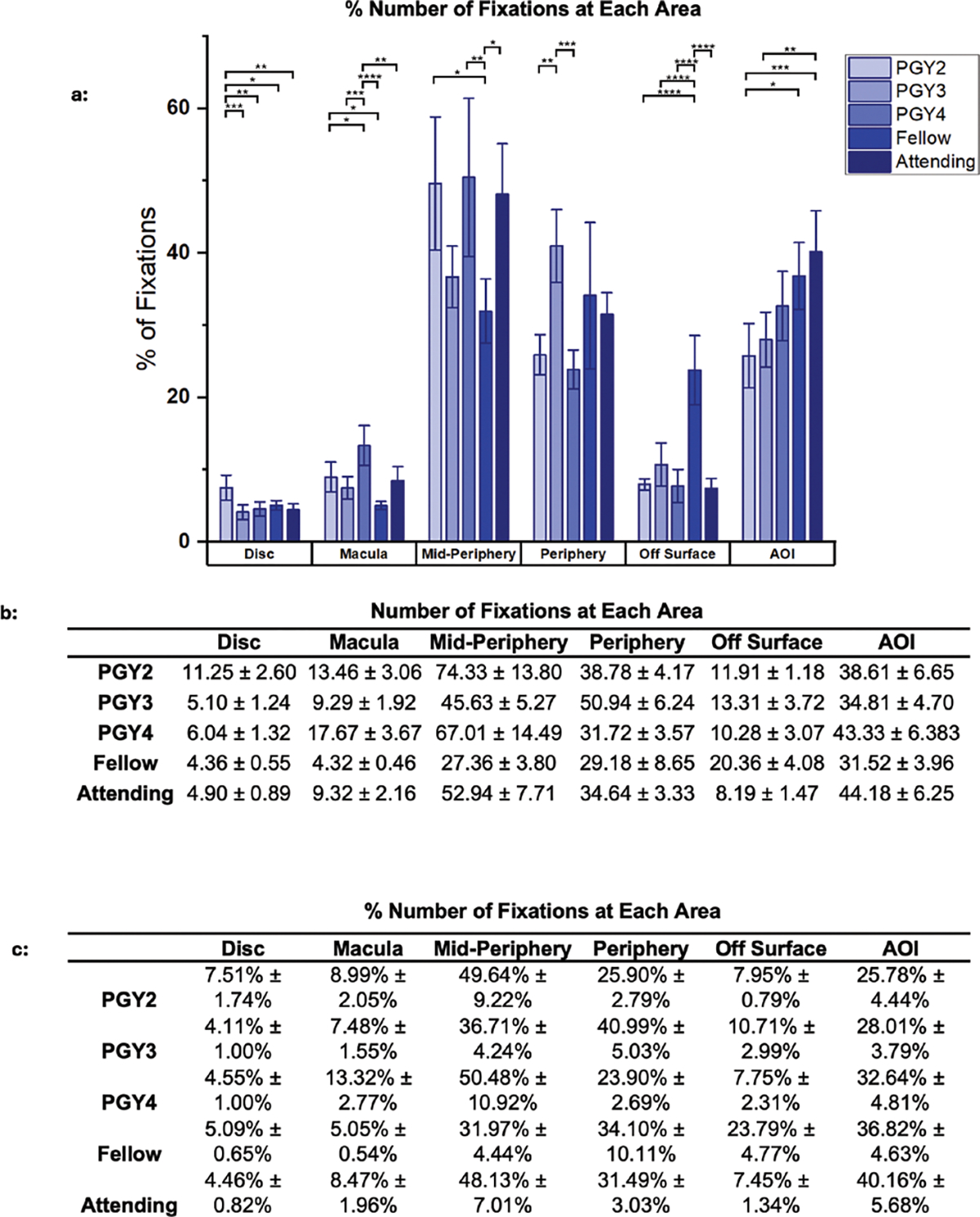
Accumulated data from each of the individual experience levels. (A) Plotted data of the % number of fixations spent on analysis in each eye location from the participant groups for between group analysis. (B) Table values of the plotted number of fixations data. (C) Table values of the % number of fixations spent on analysis in each eye location from the participant groups for between group analysis. * *P* < 0.05. ** *P* < 0.01. *** *P* < 0.001. **** *P* < 0.0001.

The next analysis compares fixation patterns on fundus images across varying levels of medical experience (Figure 4). This data revealed that attendings and PGY2s allocated a comparable number of fixations to the area of interest, with attendings averaging 44.18064 ± 6.25362 (40.16% ± 5.68%) and PGY2s close behind at 38.61052 ± 6.65131 (25.78% ± 4.44%). However, attendings distributed their fixations more evenly across all anatomical sites, dedicating 52.94 ± 7.71 (48.13% ± 7.01%) and 9.32 ± 2.16 (8.47% ± 1.96%) fixations to the mid-periphery and macula, respectively, while PGY2s focused predominantly on the vessel (mid-periphery) with the highest number of fixations at 74.33 ± 13.80 (49.64% ± 9.22%).

### VISUAL SEARCH PATTERNS

**Figure 5.**
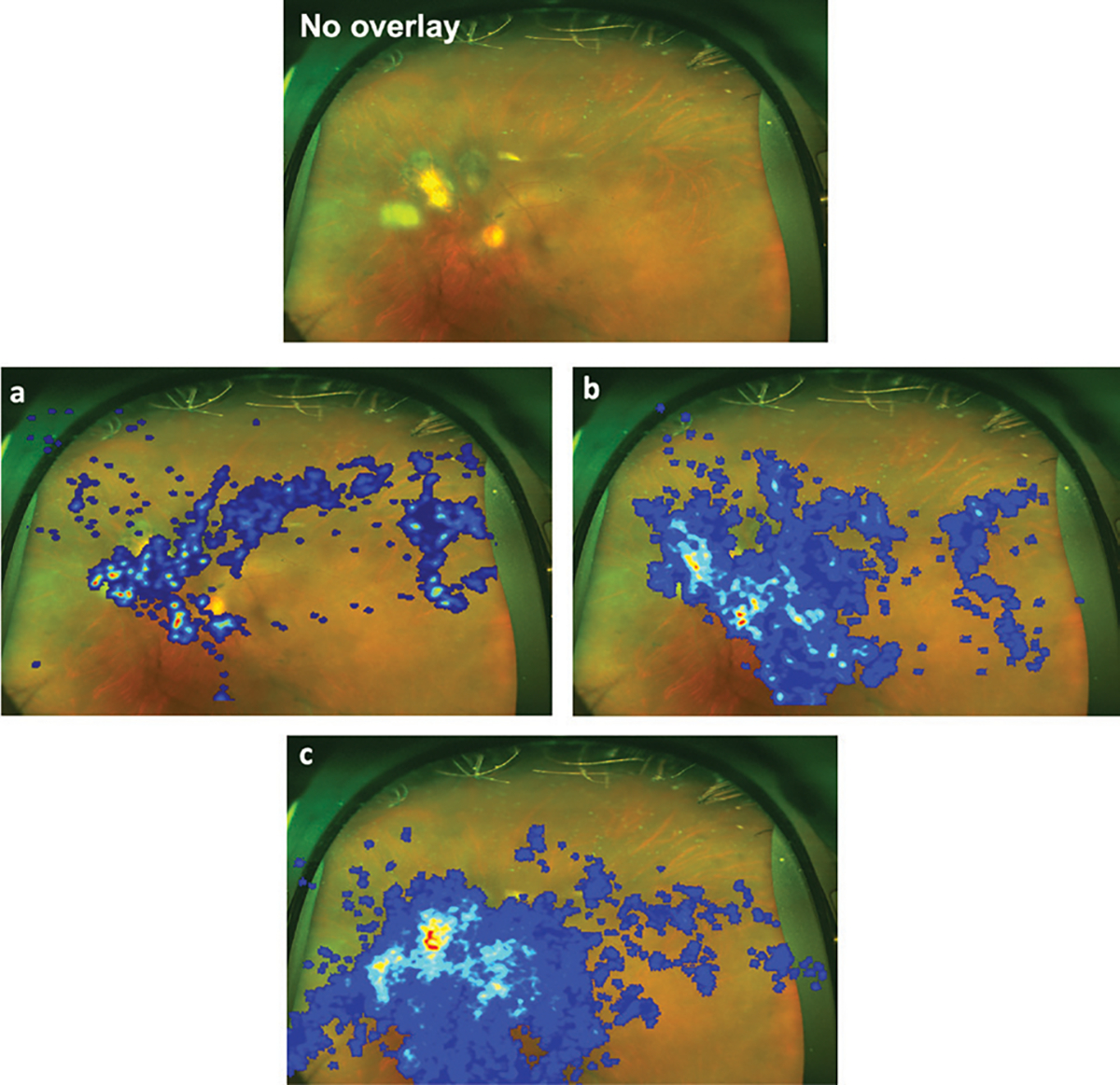
Sample gaze heat map data overlayed on top of the toxoplasmosis retinochoroiditis. (A) Postgraduate year 2 (PGY2) – junior resident. (B) Postgraduate year 4 (PGY4) – senior resident. (C) Attending vitreoretinal surgeon.

Based on criteria from existing literature, a qualitative analysis was employed to analyze gaze patterns between experience levels. A single representative image was selected to present the general findings. This image presented a case of ocular toxoplasmosis, with an area of interest containing a chorioretinal scar with associated intraocular inflammation nasal to the optic disc (Figure 5; Videos 1–3, available with this article online). The junior PGY2s’ and PGY3s’ gaze patterns have an initial sporadicity without consistent fixation on the area of interest until after repetitive scanning throughout the image. This trend is corroborated by the composite heat map data, wherein the intensity of color signifies the frequency of fixations. The PGY4s and fellows show a more concentrated focus on areas with inflammation and heightened attention to the peripheral regions of the image. Finally, the expert attending’s gaze pattern illustrates an initial concentration on the inflammatory regions and is consistently followed by a subsequent examination of the periphery. The attendings’ heat map validates these observations with intensified colors surrounding the inflammatory regions and a noticeable border of consistent color intensity, signifying systematic peripheral analysis.

## DISCUSSION

In the present study, we have observed that increased experience in analyzing retinal imaging—defined by the participants’ level of medical training, from postgraduate year residents to attending vitreoretinal surgeons—corroborated by response accuracy, is accompanied by decreased fixation durations, fewer blinks per minute, shorter saccade lengths, and a higher percentage of the image area fixated upon by more experienced clinicians. These findings contribute to a growing body of evidence indicating that visual search characteristics reflect the cognitive strategies employed by individuals at different levels of medical training.^5^

The higher fixation count observed among retina attendings aligns with past research in radiology. For instance, experts searching CT scans for enlarged lymph nodes were observed to have higher fixations than novices. This study speculates that the experts likely fixated faster than novices on these lymph nodes but deliberately sampled other areas where abnormalities may present.^3,16^ Additionally, their shorter fixation durations are consistent with their ability to rapidly process visual information.^17^ Furthermore, existing research has found that experts with these gaze characteristics use a global-focal pattern and possess an intuitive ‘gist’ recognition.^18,19^

The decrease in blink rates associated with increased experience demonstrates that experts immerse themselves in a task more, reducing the frequency of blinks to maintain visual perception.^20,21^ However, the observed increase in blink rates among PGY4 residents is an intriguing finding, possibly due to stress or fatigue factors that have been reported to increase blink rates.^22,23^ Generally, these findings are consistent with those reported in surgery and radiology, where more experienced professionals demonstrate lower blink rates and shorter blink rate durations. These studies speculate that blinking can disrupt cognitive processes and is thus suspended during periods of enhanced concentration.^24,25^

Saccade length was observed to decrease with experience, suggesting that attending physicians use a more concentrated and meticulous visual scanning of retinal images. Furthermore, these shorter saccade lengths reflect an advanced proficiency in quickly identifying salient features within an image without requiring extensive, sweeping visual search patterns.

The analysis of the fixation and time distribution data illustrates how medical professionals’ visual strategies evolve with experience. Attendings show a well-distributed pattern of fixations across various areas, while less experienced PGY2s tend to concentrate their gaze more on specific areas like the vessels, suggesting a narrower initial focus. As they gain experience, PGY3s expand their focus to include peripheral areas, with PGY4s and fellows beginning to integrate both central and peripheral views in a more strategic manner.

The heat maps from our study reinforce these findings, where attendings display a focused yet systematic and comprehensive search pattern.^26^ This mirrors trends seen across various fields like radiology, chess, and sports, where experts tend to have more focused and efficient search patterns.^17,27,28^ Furthermore, this efficiency can be attributed to “top-down” processing, which allows them to anticipate key areas of interest quickly.^29^ For example, a less experienced observer evaluating a central retinal vein occlusion might be drawn to the diffuse blood and thunder, while the experienced observer would quickly evaluate the optic disc for subtle lacy neovascularization with key patient management implications.

It is crucial to recognize that the relationship between expertise and visual cognitive processes is influenced by individual differences, task characteristics, and training practices.^17^ Nevertheless, these findings show variations in visual search strategies as a function of experience level.

Prior research comparing the search patterns of ophthalmologists and optometrists using eye-tracking technology reveals that both groups exhibit the longest fixation durations and highest fixation counts on critical regions, such as the optic disc and macula, during fundus image evaluation.^30^ Our findings align with this, showing that participants across all experience levels spent more time evaluating anatomical landmarks than the AOI. This same study reported that ophthalmologists demonstrated broader, more systematic visual sweeps, while optometrists employed shorter, point-to-point visual spans.^30^ Similarly, our study found that attendings fixated on a broader percentage of the image area compared to PGY2 residents, suggesting that experienced clinicians adopt comprehensive visual search strategies, incorporating peripheral processing and perceptual chunking. Heat map analyses in both studies corroborate these trends, with experienced practitioners systematically covering central and peripheral regions. While the prior study identified a positive correlation between fixation metrics and diagnostic accuracy, our results show that diagnostic accuracy improved with experience, correlating with decreased fixations. This is notable, especially since other research has linked prolonged review times to higher diagnostic error rates.^31^ These parallels suggest that the systematic visual strategies developed by ophthalmologists and optometrists are mirrored in the progression of medical trainees.

Research in radiology and pathology has proven that there are significant differences between the visual search patterns of experts and trainees. Experts in these fields are known to employ a global-focal search, which allows for a more efficient diagnosis of abnormalities.^3,16^ This search pattern involves first a quick, comprehensive view of the image, followed by a slower, focal search of the abnormalities.^32,33^ This finding corroborates our data, where expert ophthalmologists employed search patterns similar to that of a global-focal pattern.

While the disc-macula-vessel-periphery sequence is already a well-established method in clinical training, our results suggest potential opportunities to optimize this strategy by incorporating additional elements observed in expert-level searches. Specifically, our study highlights the importance of incorporating peripheral processing and perceptual chunking into the training process. For instance, attendings adhered to the disc-macula-vessel-periphery sequence and employed multiple outward sweeps through each quadrant, ensuring a thorough peripheral examination. This suggests that teaching residents to integrate repeated radial sweeps and peripheral scanning alongside the standard sequence may enhance diagnostic accuracy. Furthermore, our data highlight the role of fixation efficiency, with experts demonstrating shorter fixation durations and fewer overall fixations, indicating rapid visual information processing. To optimize the disc-macula-vessel-periphery method, training programs could potentially incorporate exercises that promote fixation efficiency, such as timed diagnostic challenges or simulations that reward accurate, rapid assessments.

This study has several limitations that should be acknowledged. First, the sample size remains relatively small and is selected from a single institution, which may limit the generalizability of our findings. While the homogeneity of our sample helps control for variability, it may not fully capture the diversity of ophthalmology trainees or practicing attendings. Future studies incorporating larger and more diverse populations across multiple institutions would be beneficial in determining whether our findings hold true in broader clinical settings. Second, the use of a limited set of retinal images may not fully capture the range of pathologies encountered in clinical practice. Third, we did not objectively measure the emotional or cognitive load of participants, which could provide further insight into visual search patterns and diagnostic performance. Additionally, individual variations in prior experience, such as pre-residency exposure to retinal imaging, may blur the distinctions between senior and junior residents; however, experience within groups was generally similar.

## CONCLUSION

While this study highlights distinct scanning patterns between experienced and less experienced examiners, it is important to emphasize that visual search strategies alone do not substitute for the extensive clinical experience required for expertise in retinal image interpretation. Expertise in ophthalmology, as in other domains of medicine, is cultivated through years of deliberate practice, case exposure, and real-world decision making. Longitudinal research is needed to validate these findings and suggest specific search patterns that can complement traditional training. By addressing these limitations in future research, the implementation of standardized viewing techniques can be studied. Furthermore, eye-tracking technology could be used in such training programs to provide feedback on residents’ gaze patterns and help them develop more effective search strategies.^34^

## Figures and Tables

**Figure A:**
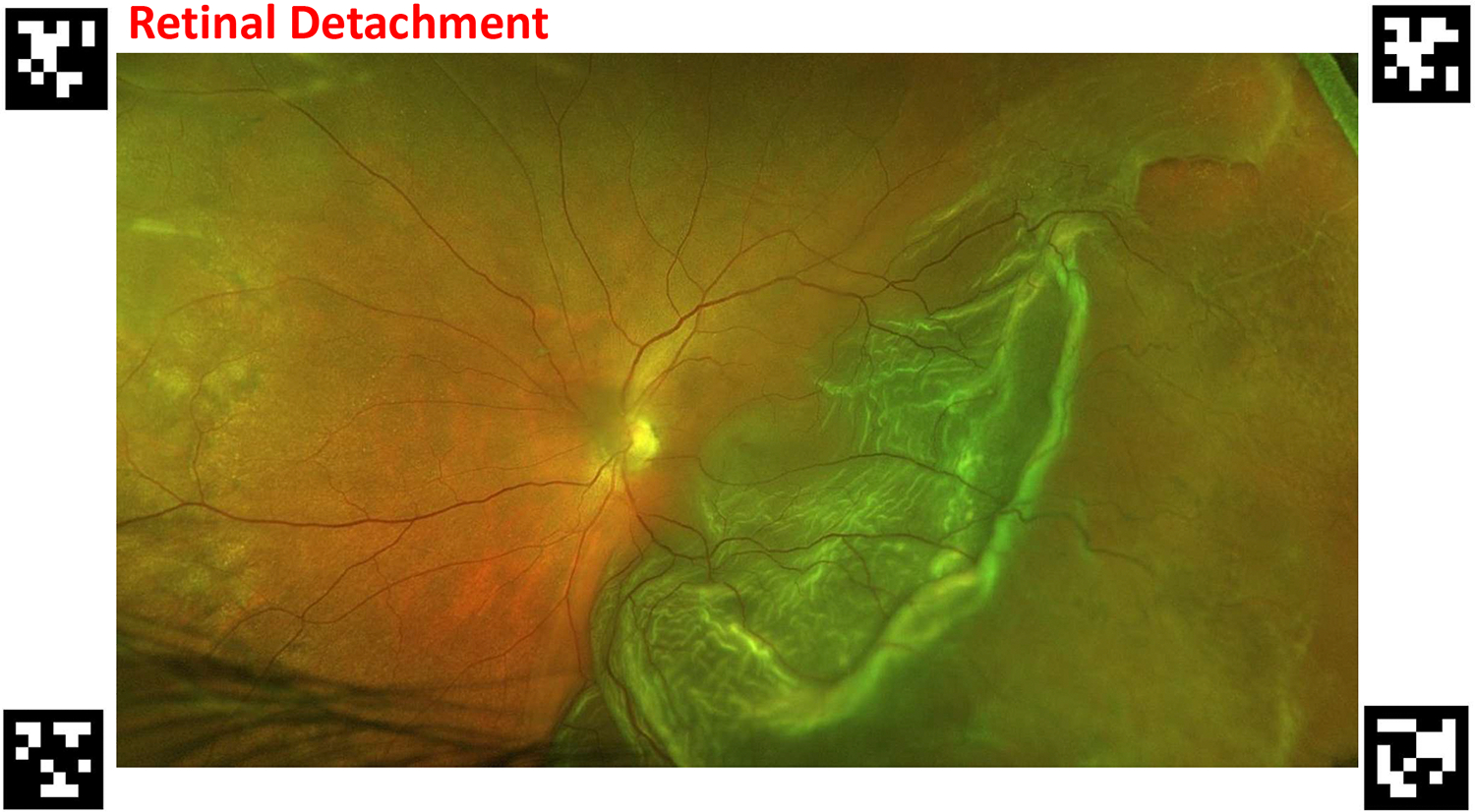

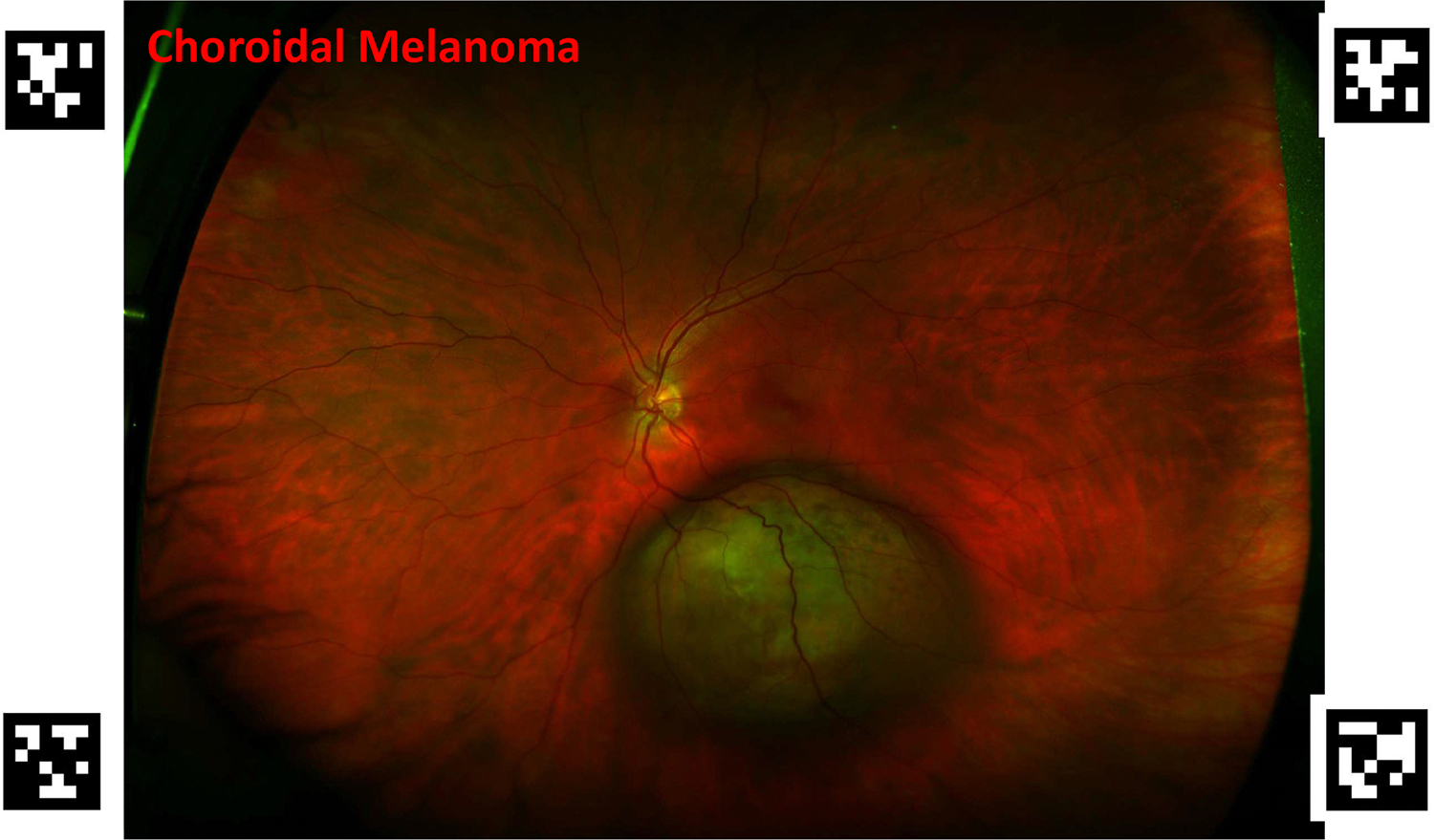

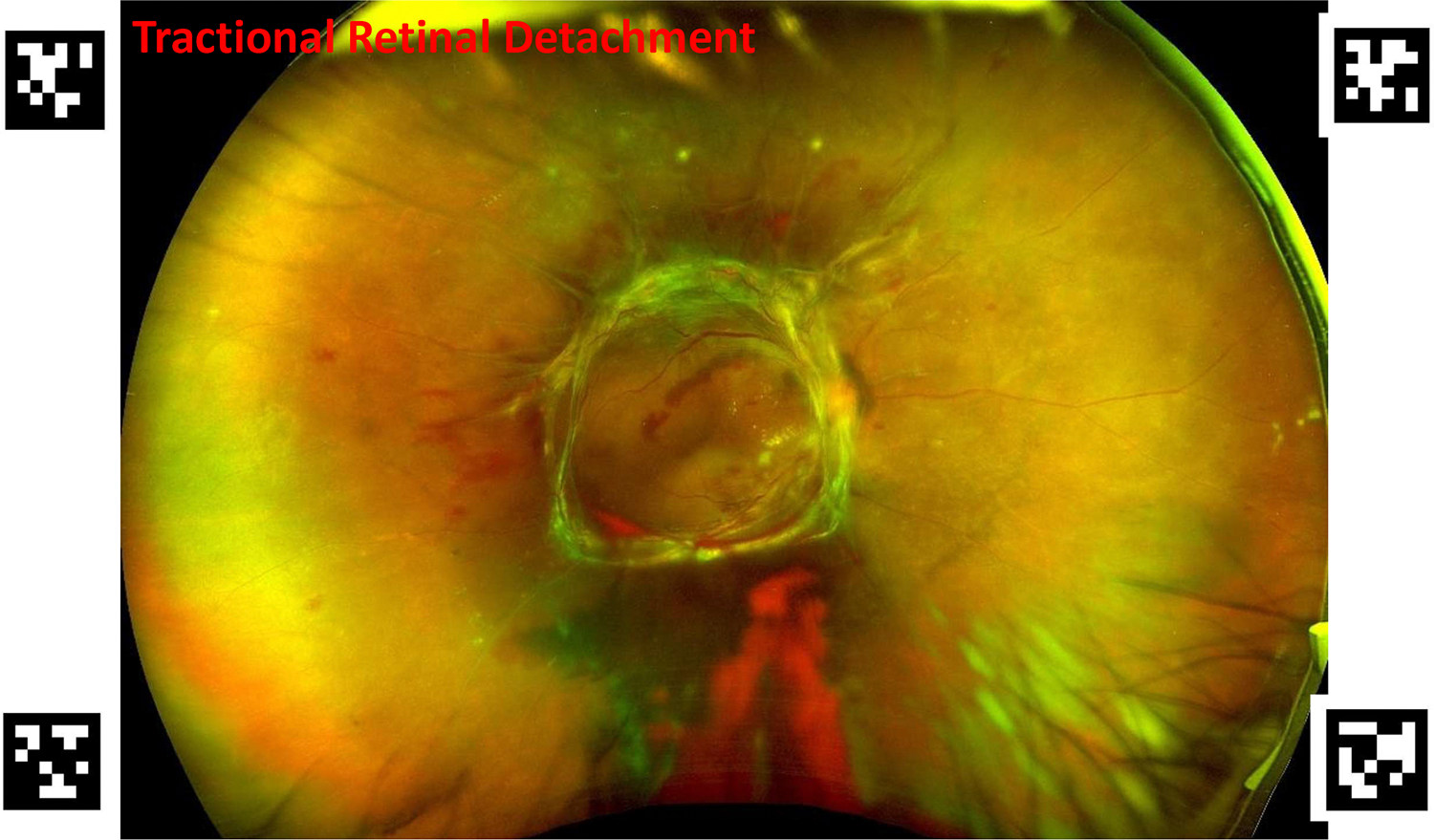

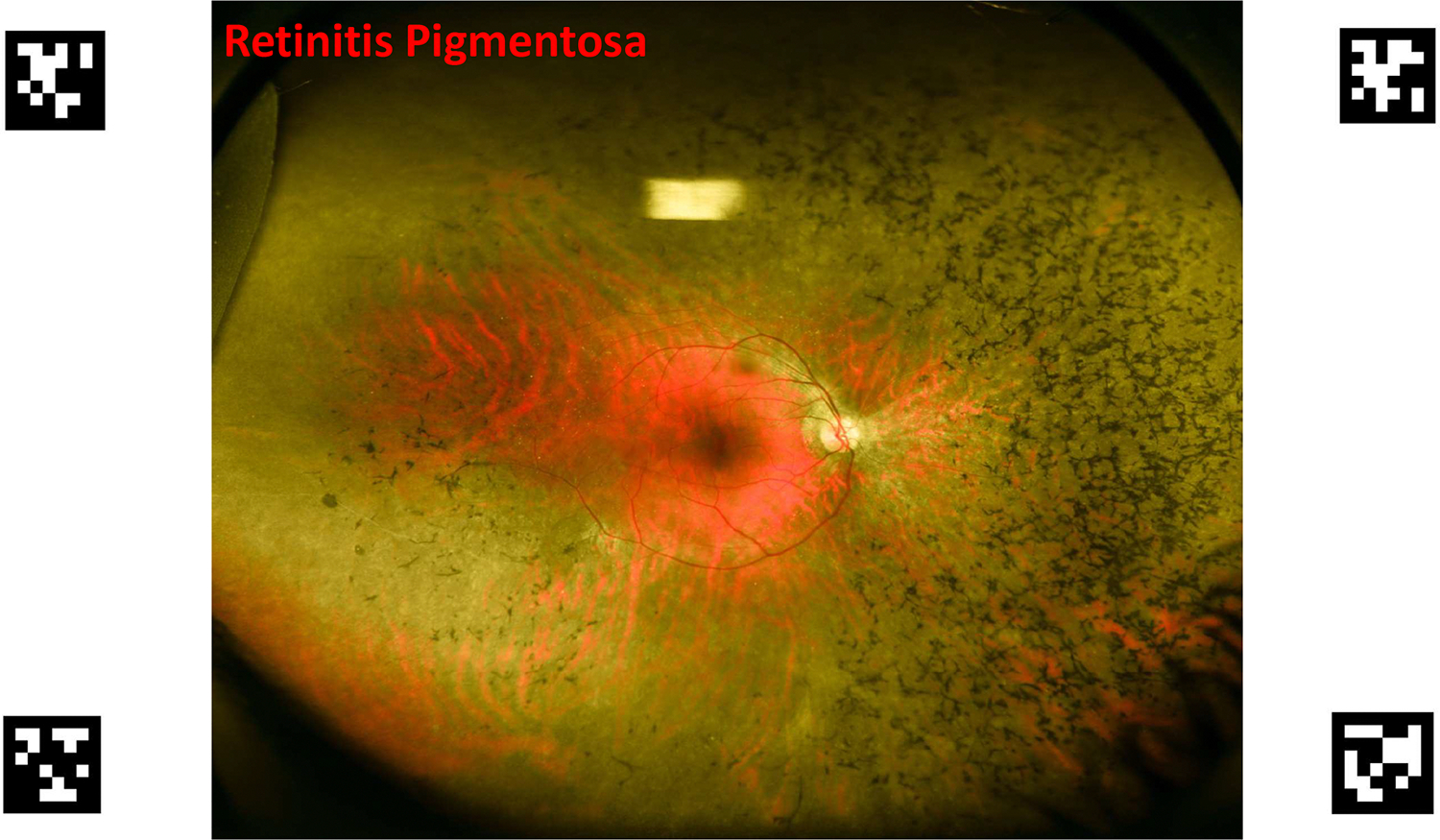

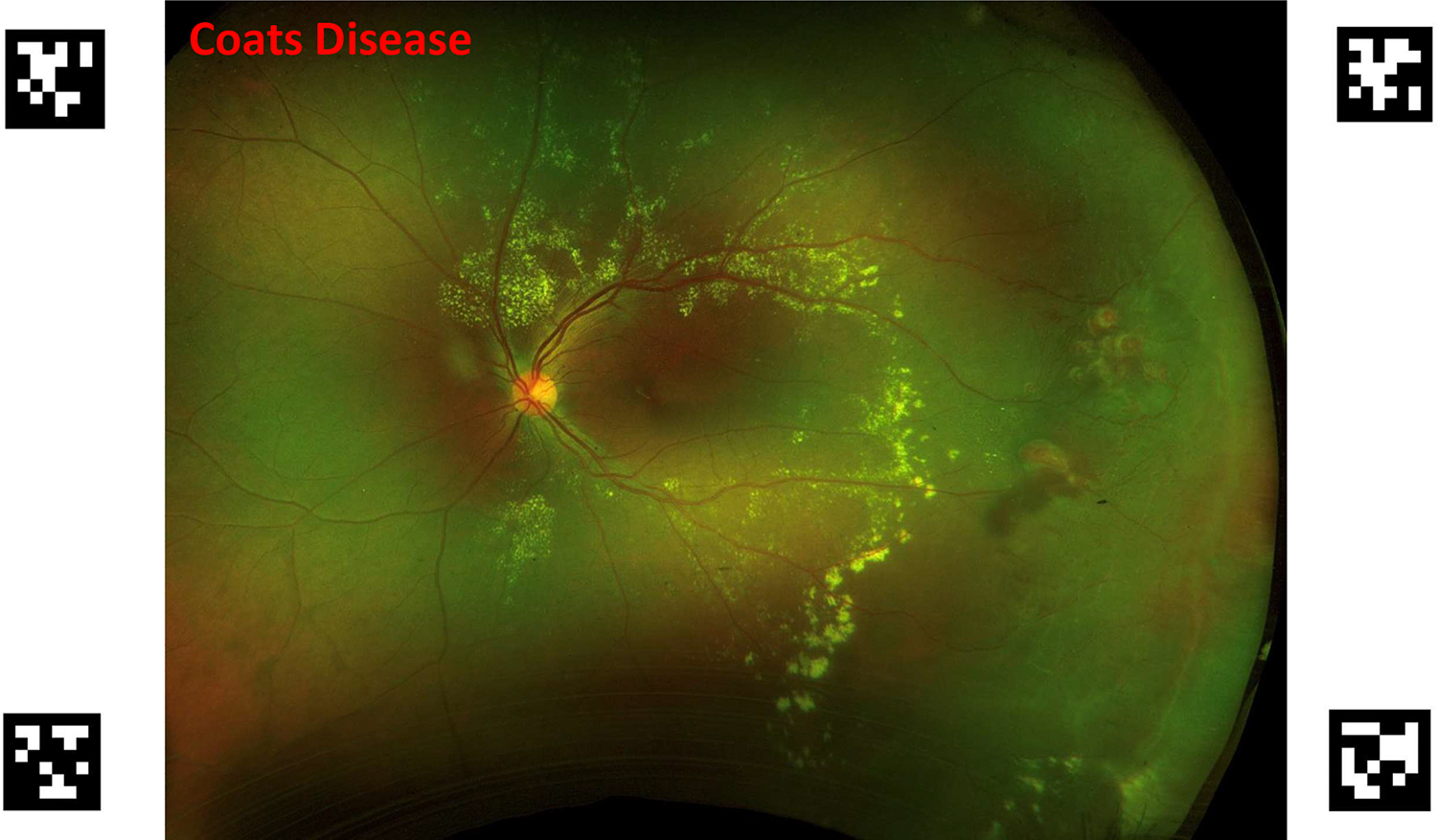

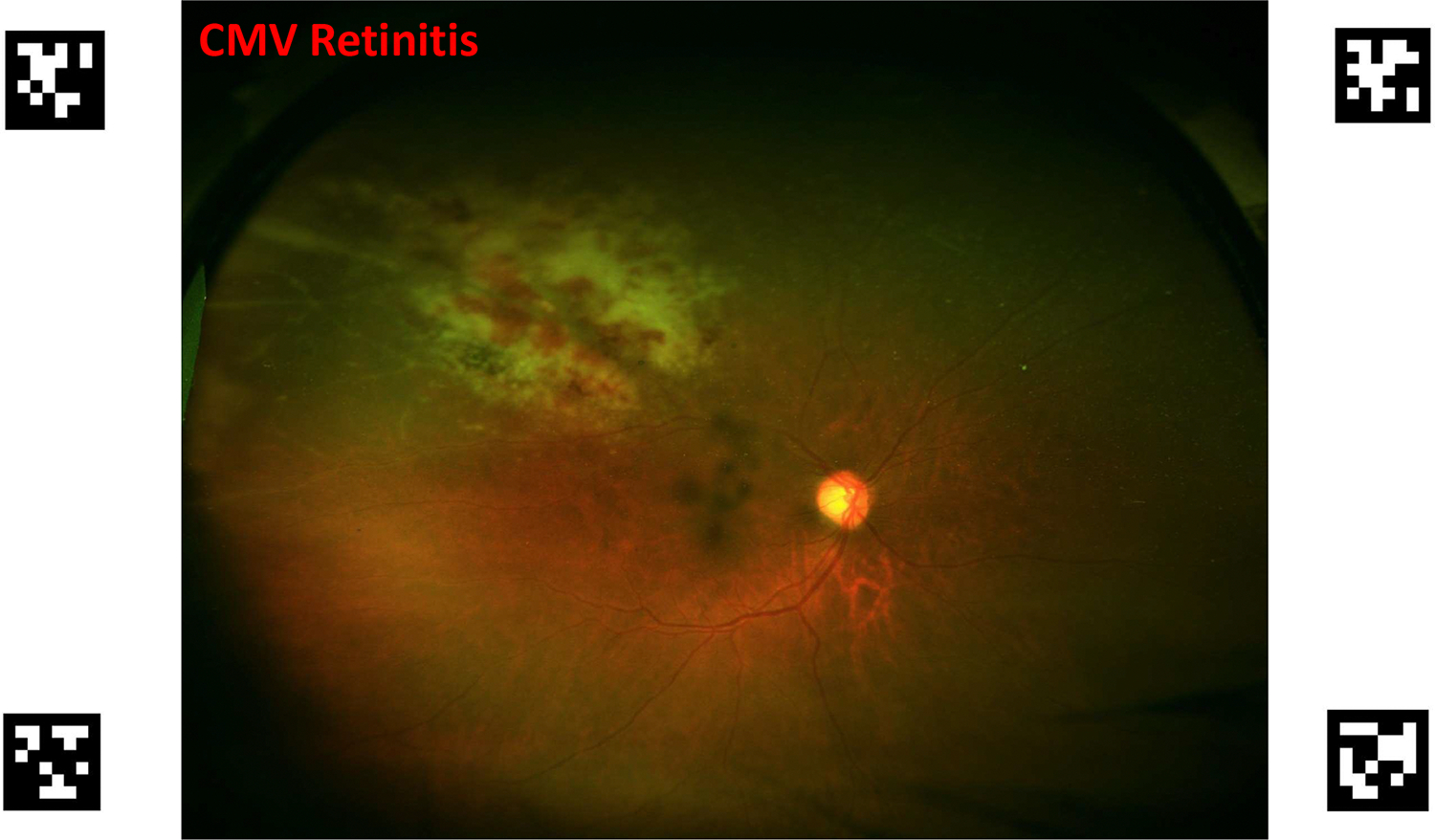

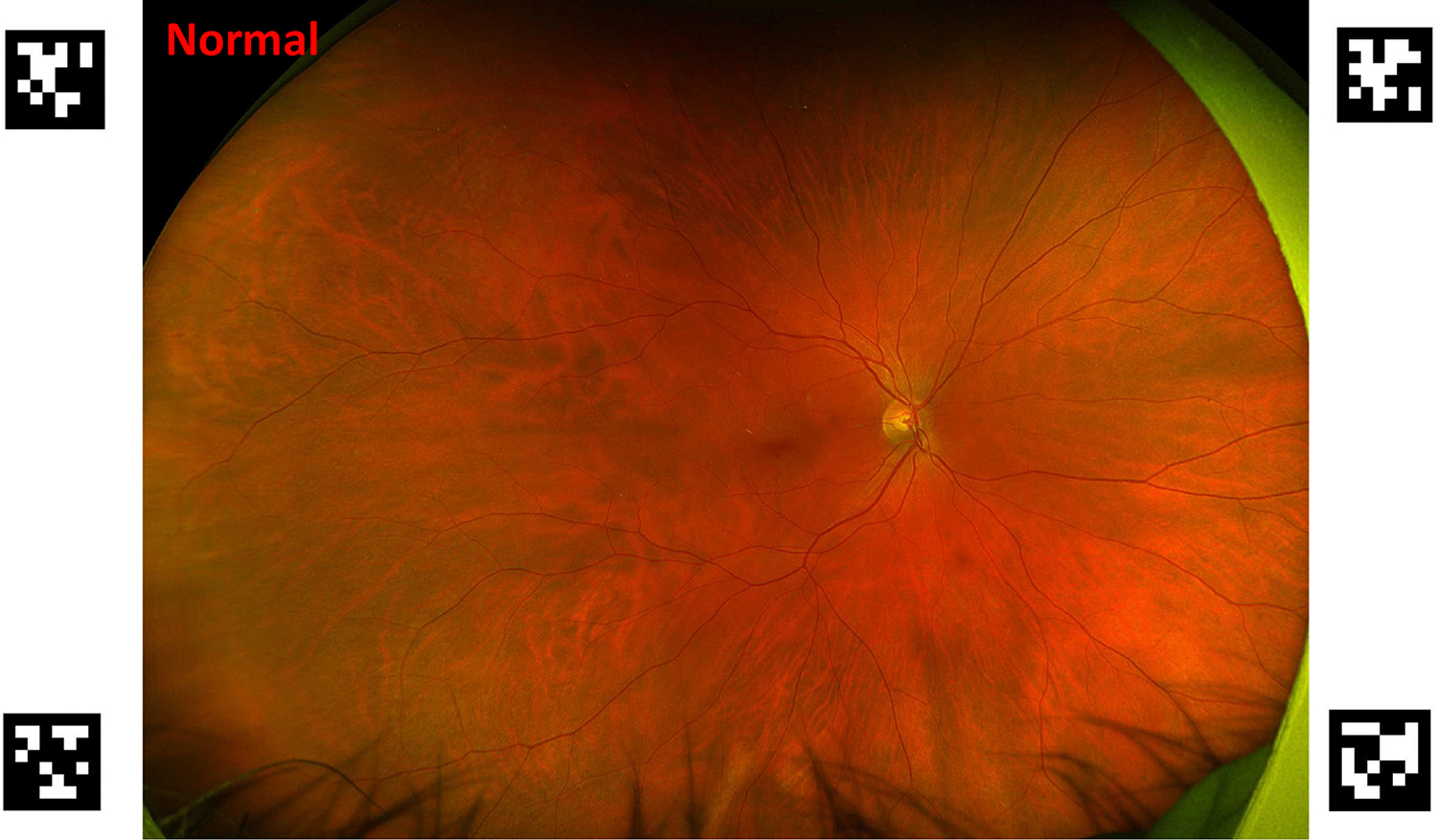

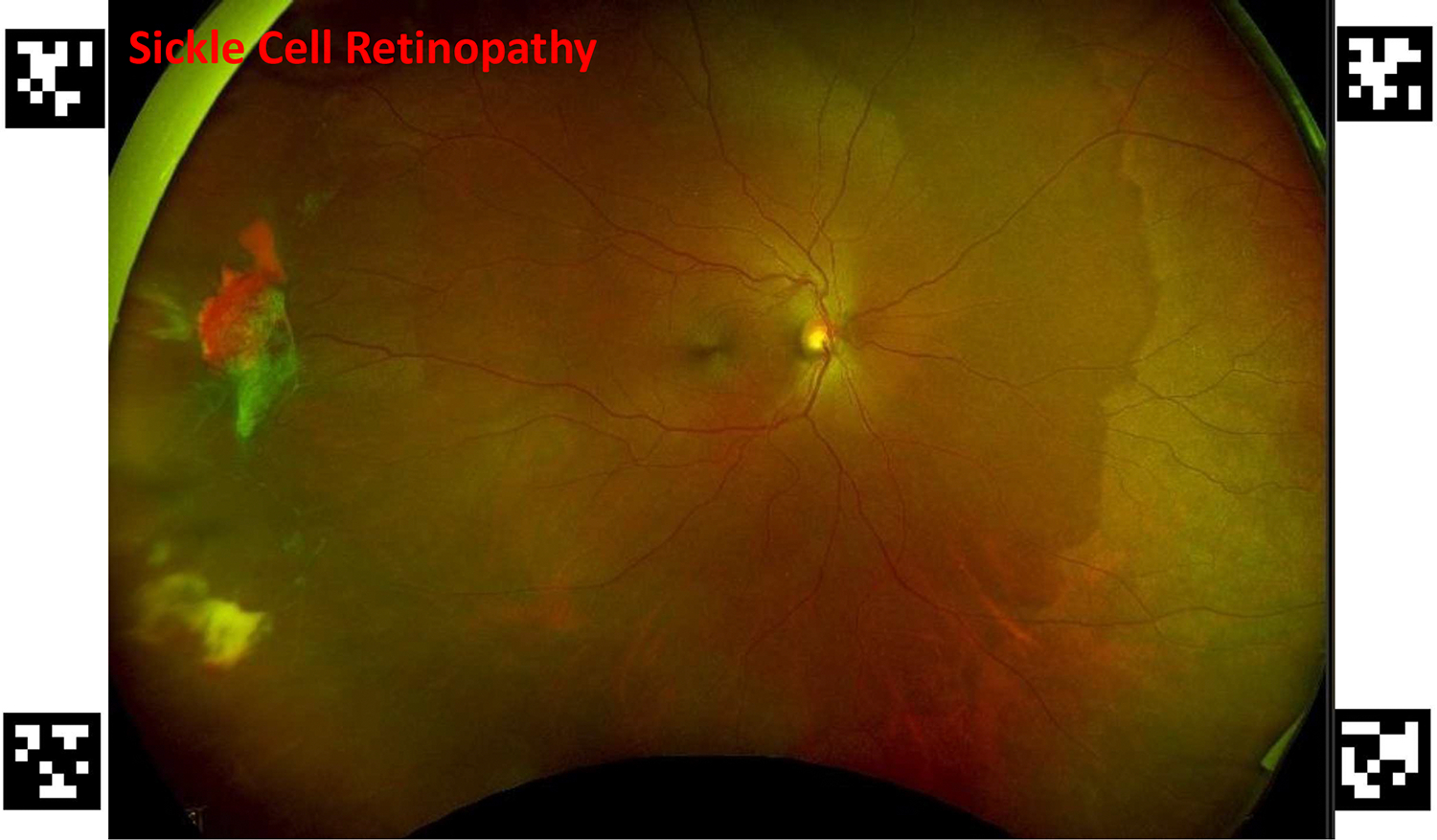

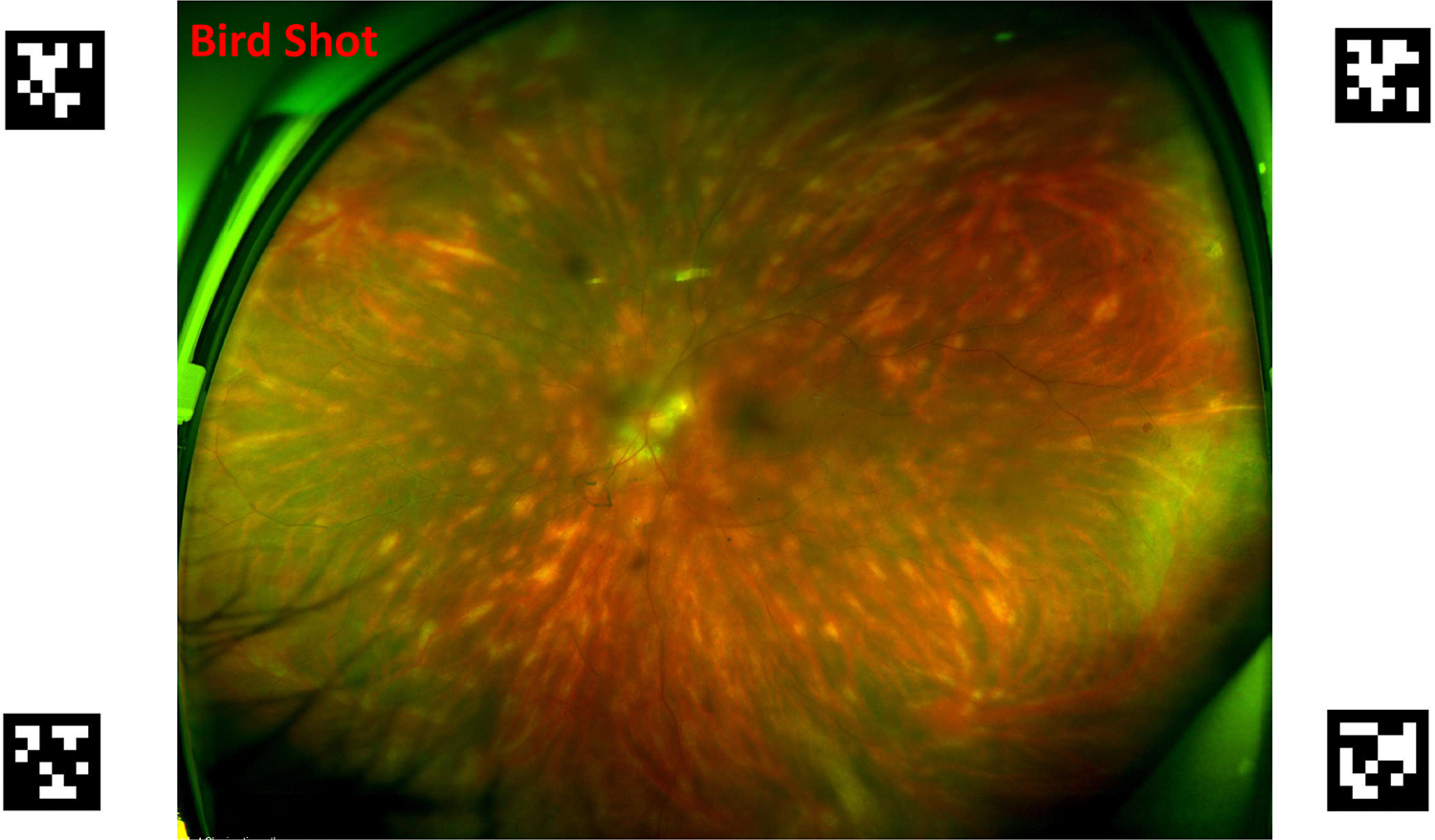

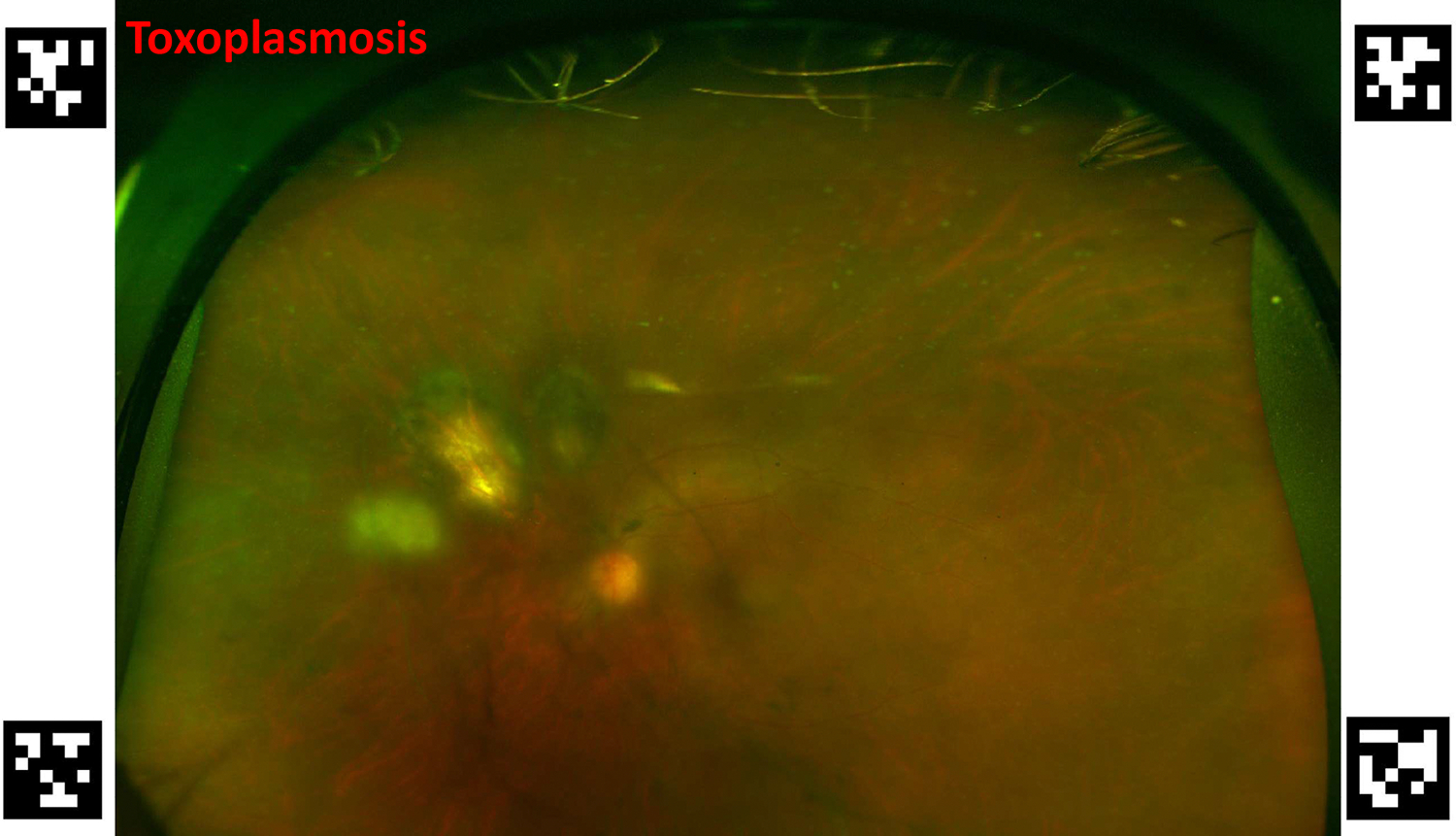

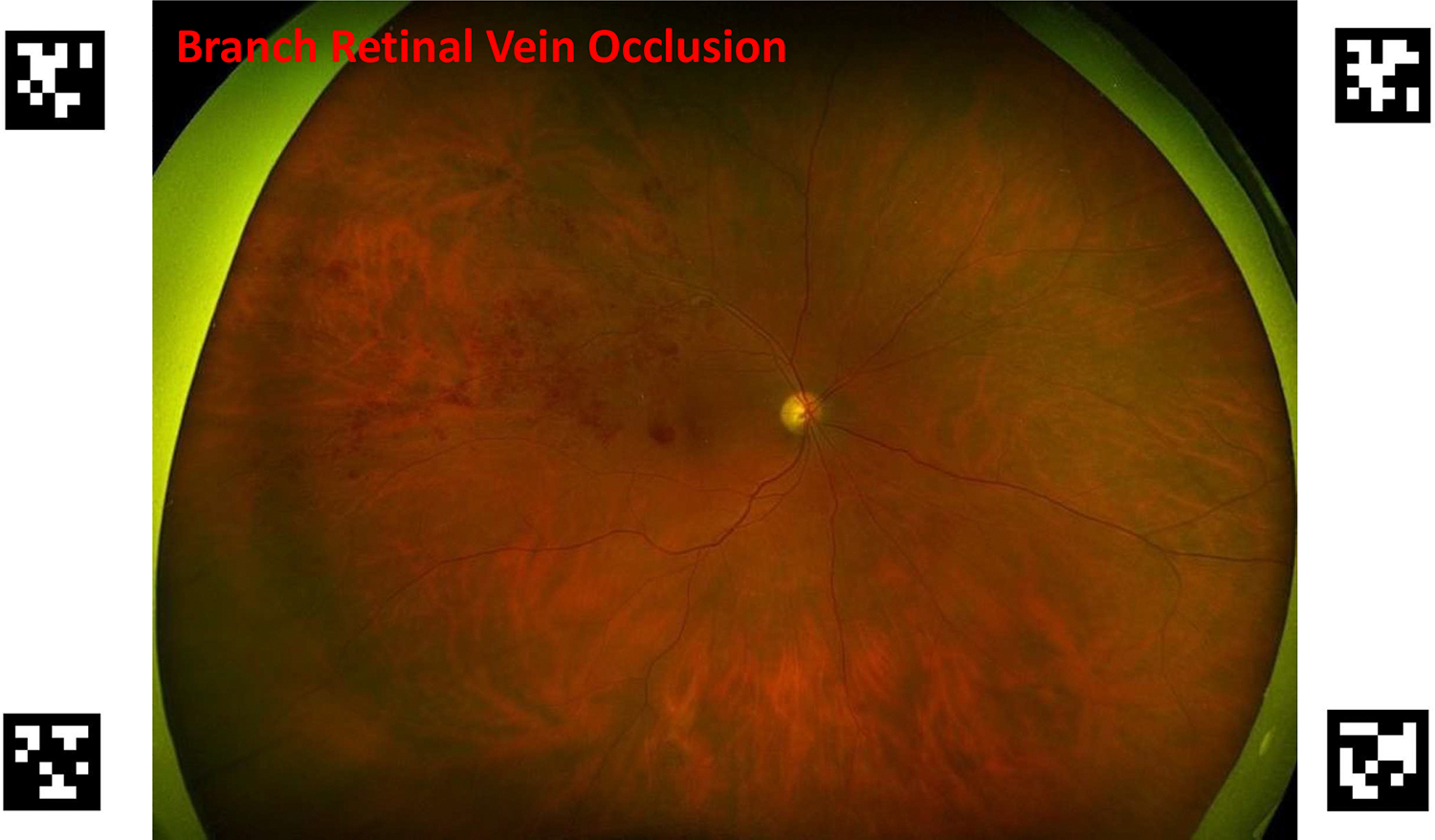

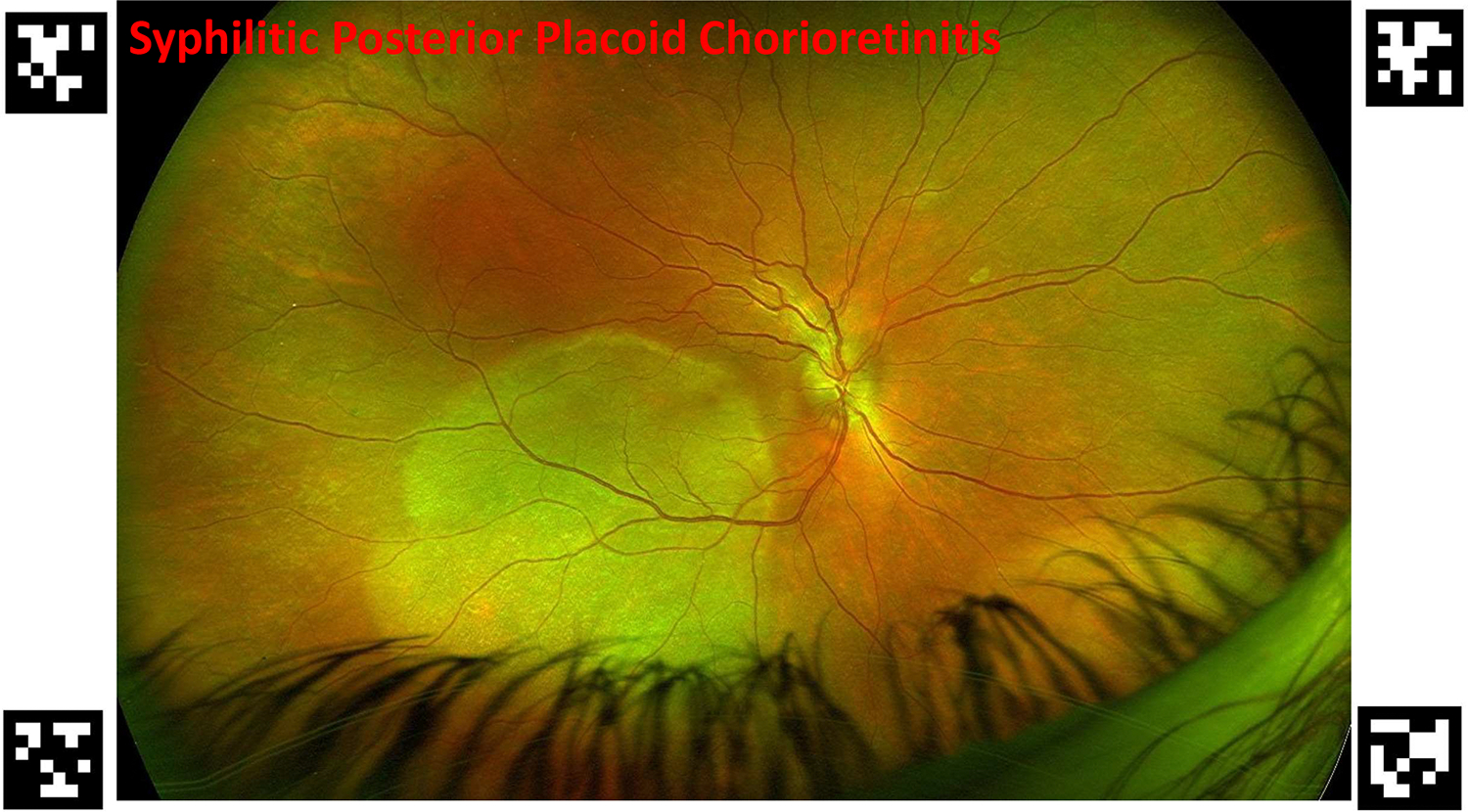

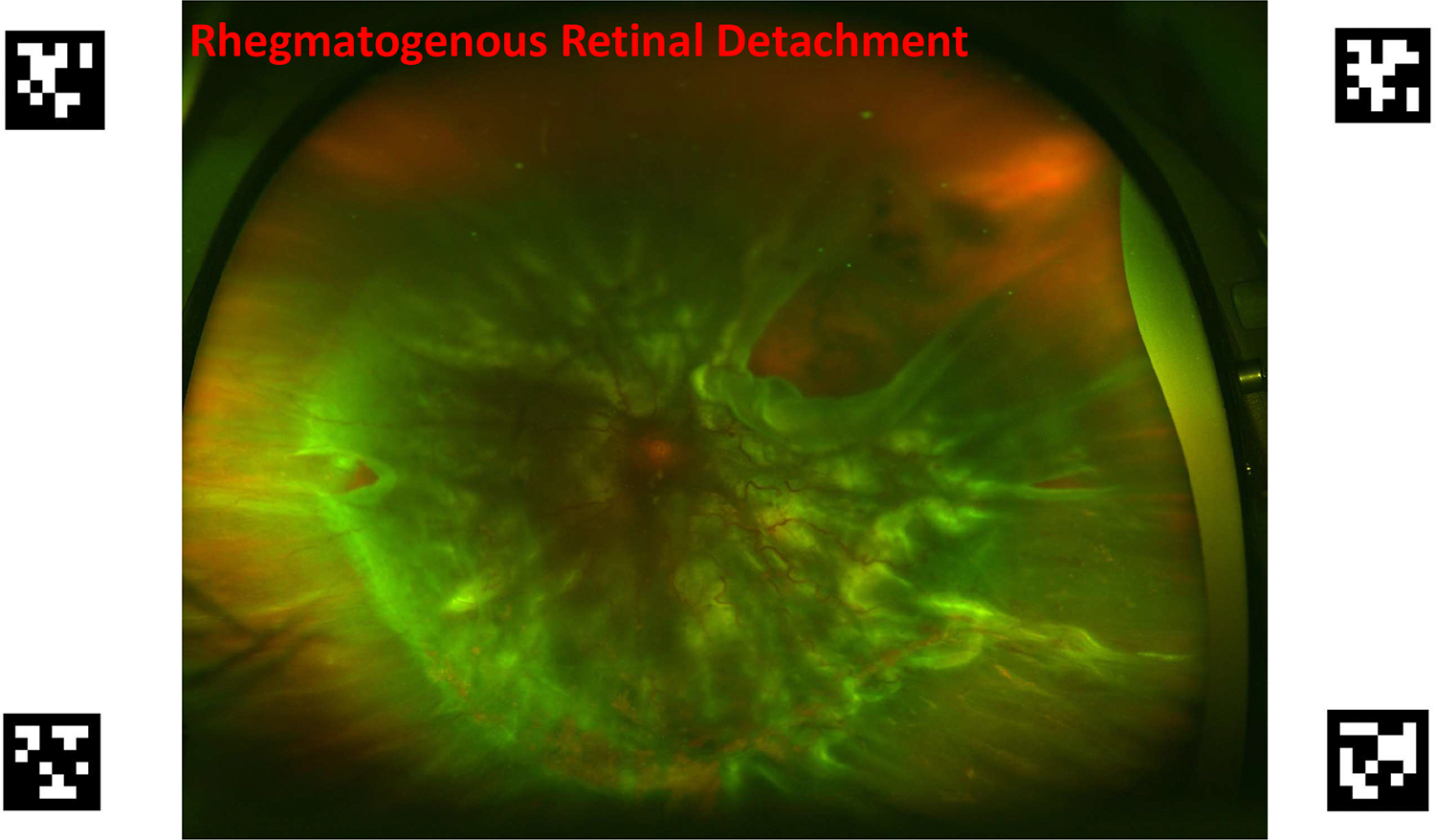

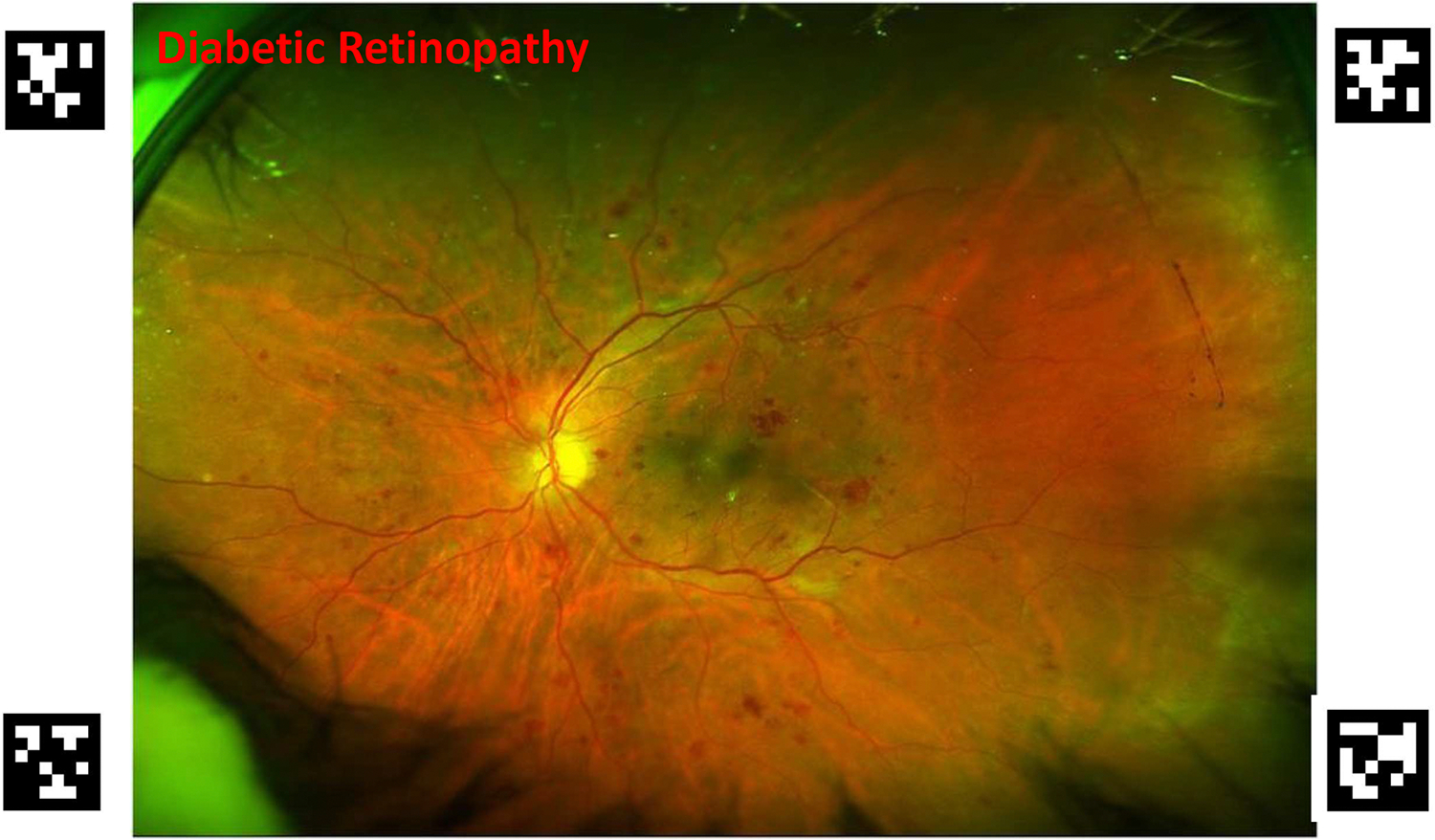

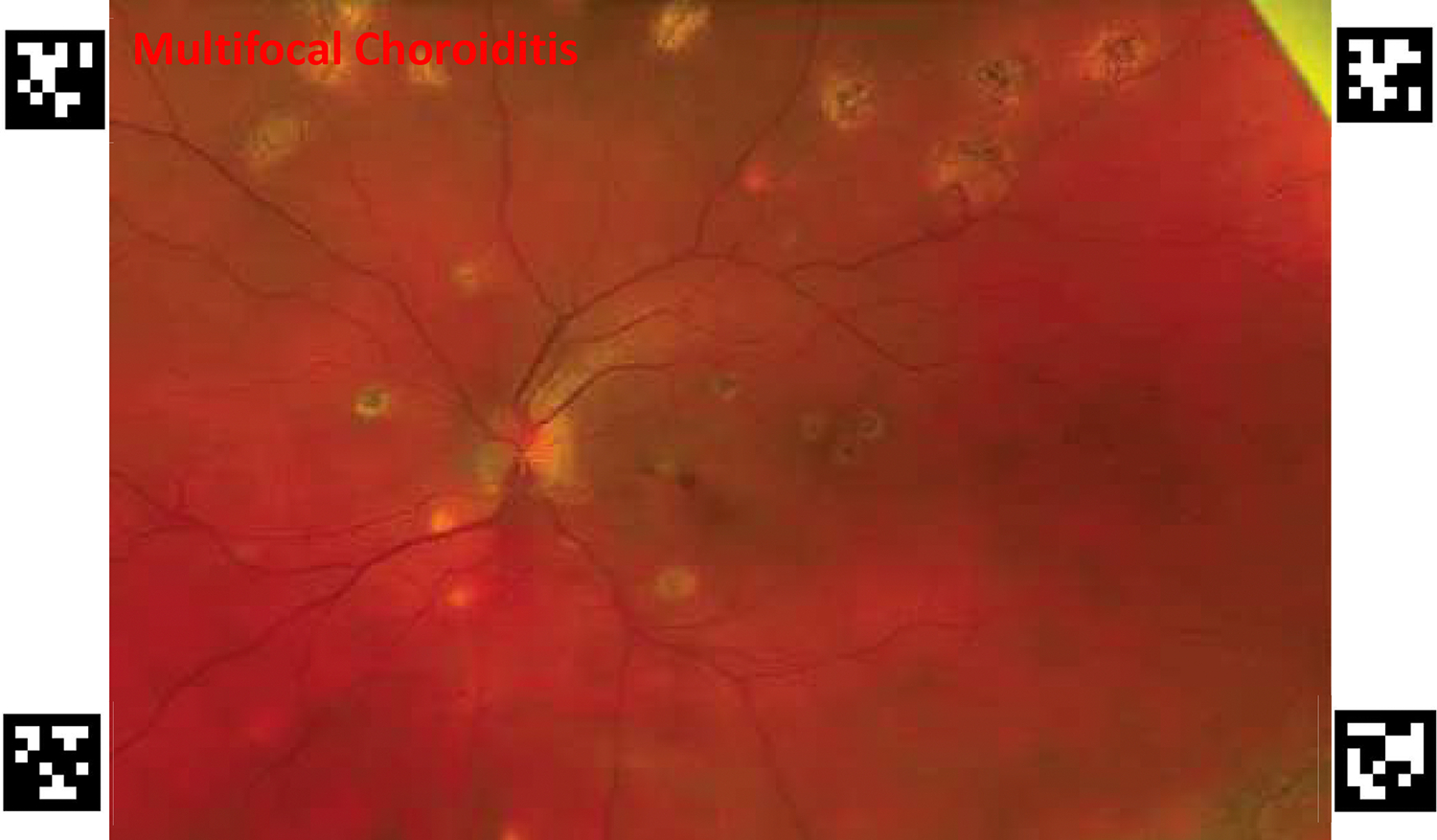
Fundus images.

**Video 1:** 

**Video 2:** 

**Video 3:** 

**TABLE 1:** Participant Demographics

Characteristic	PGY2	PGY3	PGY4	Fellow	Attending
Number of participants (*n*)	5	5	5	5	5
Age (years, mean ± SD)	29.4 ± 0.5	30.4 ± 0.4	31 ± 0.6	32.2 ± 0.4	38.6 ± 2.2
Years of experience	1 ± 0	2 ± 0	3 ± 0	4.4 ± 0.5	9.2 ± 0.7
Men/Women	3/2	4/1	2/3	3/2	3/2

PGY2 = postgraduate year 2; PGY3 = postgraduate year 3; PGY4 = postgraduate year 4; SD = standard deviation.
